# Deciphering Genotype-By-Environment Interaction for Target Environmental Delineation and Identification of Stable Resistant Sources Against Foliar Blast Disease of Pearl Millet

**DOI:** 10.3389/fpls.2021.656158

**Published:** 2021-05-17

**Authors:** S. Mukesh Sankar, S. P. Singh, G. Prakash, C. Tara Satyavathi, S. L. Soumya, Yashpal Yadav, L. D. Sharma, A. R. Rao, Nirupma Singh, Rakesh K. Srivastava

**Affiliations:** ^1^Division of Genetics, ICAR-Indian Agricultural Research Institute, New Delhi, India; ^2^Division of Plant Pathology, ICAR-Indian Agricultural Research Institute, New Delhi, India; ^3^ICAR-All India Coordinated Research Project on Pearl Millet, Jodhpur, India; ^4^CCS Haryana Agricultural University, College of Agriculture, Bawal, India; ^5^Rajasthan Agricultural Research Institute, Jaipur, India; ^6^CABin, ICAR-Indian Agricultural Statistical Research Institute, New Delhi, India; ^7^International Crops Research Institute for the Semi-Arid Tropics, Patancheru, India

**Keywords:** pearl millet, *Magnaporthe*, blast disease, genotype-environment interaction, heritability, GGE biplots, mega-environments, mixed-model analysis

## Abstract

Once thought to be a minor disease, foliar blast disease of pearl millet, caused by *Magnaporthe grisea*, has recently emerged as an important biotic constraint for pearl millet production in India. The presence of a wider host range as well as high pathogenic heterogeneity complicates host–pathogen dynamics. Furthermore, environmental factors play a significant role in exacerbating the disease severity. An attempt was made to unravel the genotype-by-environment interactions for identification and validation of stable resistant genotypes against foliar blast disease through multi-environment testing. A diversity panel consisting of 250 accessions collected from over 20 different countries was screened under natural epiphytotic conditions in five environments. A total of 43 resistant genotypes were found to have high and stable resistance. Interestingly, most of the resistant lines were late maturing. Combined ANOVA of these 250 genotypes exhibited significant genotype-by-environment interaction and indicated the involvement of crossover interaction with a consistent genotypic response. This justifies the necessity of multi-year and multi-location testing. The first two principal components (PCs) accounted for 44.85 and 29.22% of the total variance in the environment-centered blast scoring results. Heritability-adjusted genotype plus genotype × environment interaction (HA-GGE) biplot aptly identified “IP 11353” and “IP 22423, IP 7910 and IP 7941” as “ideal” and “desirable” genotypes, respectively, having stable resistance and genetic buffering capacity against this disease. Bootstrapping at a 95% confidence interval validated the recommendations of genotypes. Therefore, these genotypes can be used in future resistance breeding programs in pearl millet. Mega-environment delineation and desirability index suggested Jaipur as the ideal environment for precise testing of material against the disease and will increase proper resource optimization in future breeding programs. Information obtained in current study will be further used for genome-wide association mapping of foliar blast disease in pearl millet.

## Introduction

Pearl millet [*Pennisetum glaucum* (L.) R. Br.] is a major climate resilient cereal crop that is cultivated extensively on resource-poor marginal lands of arid and semiarid regions of Asia and sub-Saharan Africa (Anuradha et al., 2017). It forms a source of food and fodder and ensures food and nutritional security to the inhabitants who are practicing low-input agriculture (Pankaj et al., 2020). Being a “Nutri-cereal” and thriving well in any cropping system, it shows a crucial role in defeating malnutrition and improving the socioeconomic status of resource-poor farmers (Govindaraj et al., 2020). Foliar blast (FB) disease of pearl millet caused by the fungus *Pyricularia grisea* (Cooke) Sacc. [Teleomorph: *Magnaporthe grisea* (Herbert) Barr], a disease of negligible importance in past years, has become a severe menace to successful pearl millet cultivation worldwide (Sharma et al., 2018). It is widespread in the different pearl millet-growing ecologies of India but became a very serious threat in both A_1_ (includes rainfed areas of western Rajasthan, as well as parts of Gujarat and Haryana, where annual precipitation is anticipated to be < 400 mm and pearl millet productivity is supposed to be less than 100 kg/ha) and A zones (includes North Indian regions excluding regions covered in A_1_ zones with an annual rainfall of more than 400 mm), where early- to medium-maturing cultivars are preferred (AICPMIP, 2020). In fact, the disease has reached a critical stage that necessitates a multifaceted approach to its effective management (Sharma et al., 2020).

**FIGURE 1:**
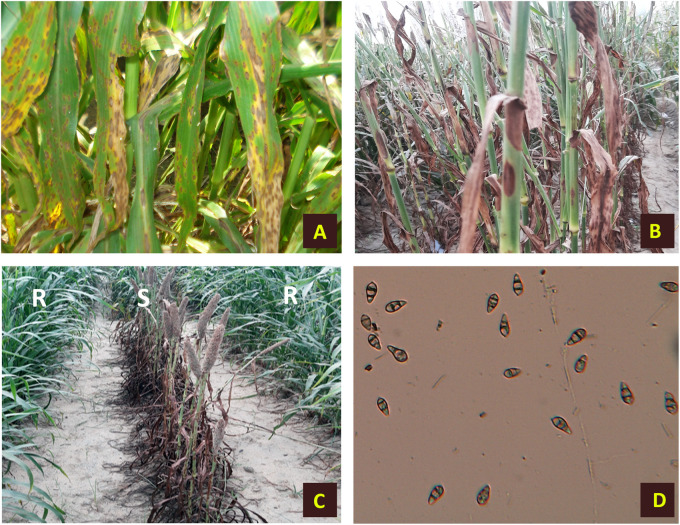
Symptoms of foliar blast on infected pearl millet plants. **(A)** Infected leaves. **(B)** Sheath and stem infection. **(C)** Infected plants along with resistant lines. **(D)** Conidia of *Magnaporthe grisea*.

The disease starts out as a small speck or lesion that grows larger and necrotic, causing widespread chlorosis and premature death of young leaves (Figure 1). Lesions can appear as diamond-shaped white to gray or reddish-brown lesions near the leaf tips or margins, or both with reddish to brown borders that extend down and may enlarge, coalesce, and kill entire leaves. During humid weather, particularly with dense plant stands, this disease becomes more severe. *M. grisea* is a seed-borne fungus that often survives in the soil/leaf debris as chlamydospores or free saprophytic mycelium, providing a source of primary inoculums. Repeated infection in a single crop season happens through the dissemination of asexual spore called conidia. FB on pearl millet was observed to be inversely related to days to maturity, green and dry fodder yield, seed yield per plant, and digestive dry matter, influencing crop productivity and quality (Nayaka et al., 2017).

Foliar blast in pearl millet is a multi-cycle disease, and usually crop is grown by resource-poor farmers. Thus, chemical control with repeated sprays in one crop season is not practically feasible. The development of FB-resistant cultivars is a major thrust area for the pearl millet research and development sector worldwide, as it offers an economic and eco-friendly option for managing the disease. However, due to a limited knowledge of its inheritance (Gupta et al., 2012 and Singh et al., 2018), race specificity (Sharma et al., 2021), and the rapid shift in pathogenicity of the blast fungus, as well as a scarcity of stable resistance in exotic or adapted germplasm (Sharma et al., 2020), progress in transmitting stable resistance to commercial cultivars has been slow. Another most important challenge is poor repeatability in field-plot and greenhouse ratings due to genotype-by-environment interactions (GEIs) (Prakash et al., 2016). The role of genotype × environment (G × E) interaction is also crucial for the eventual appraisal of sources of durable resistance (Singh et al., 2020). Thus, an identification of resistance source against FB in pearl millet, followed by an appraisal of the durability of resistance and its utilization in resistance breeding programs, is necessary.

Delineation of target testing environment that has good discrimination power, representativeness, and high desirability index is also indispensable for facilitating proper selection of resistance sources as well as curtailing the use of non-informative testing locations, thus reducing the cost of multi-location trials (MLTs). Previous reports have stated that genotype and identification of a testing location would be meaningful within a mega-environment (ME) (Yan et al., 2007). Data collected over years are crucial for defining an ME and improving breeding efficiency (Yan and Holland, 2010). Several statistical tools are available for establishing the role of GEI in the identification of desirable genotypes with specific and broad-spectrum adaptability over different locations (Yan and Kang, 2003). The GGE (genotype plus GEI) biplot methodology usually applies the concept of indirect selection, removes the main effect of the environment, considers only the genotypic main effect with the GEI effect in MLT datasets, and represents the result in graphical mode (Yan et al., 2000). Based on different scaling methods, various forms of GGE biplot have been developed. Among these, the heritability-adjusted GGE (HA-GGE) biplot is the most logical and precise method for the identification of genotypes and test environments (Yan and Holland, 2010).

However, information on the identification of durable FB resistance sources based on multi-environment data is scanty. Therefore, in the present study, we employed the HA-GGE biplot method to evaluate the effects of genotype, environment, and GEI for FB resistance by deploying multi-location and multi-year datasets for detecting ideal genotypes with durable resistance. The recommendation of genotypes for a specific environment was corroborated by bootstrapping at the 95% confidence limit (CL). Additionally, test environments were evaluated in terms of discrimination power, representativeness, and desirability index, followed by ME detection, to exclude redundant testing locations and to minimize the cost incurred on future evaluation programs.

## Materials and Methods

### Plant Material and Multi-Environment Field Trials

The experimental materials include a set of diversity panel, which is a subset of PMiGAP (Pearl Millet inbred Germplasm Association Panel). It is denoted as “G” followed by serial number. This panel is composed of 250 accessions collected from over 20 different countries representing the global genetic diversity of pearl millet. The panel includes inbred lines, landraces, released cultivars, germplasm accessions, and advanced breeding lines. These genotypes were evaluated for FB disease under natural epiphytotic conditions in an alpha lattice design with two replications. Supplementary Table 1 represents the list of genotypes along with details such as subspecies, botanical variety, market type, origin, and pedigree. The panel was evaluated at IARI-New Delhi (28°70′N, 76°58′E, 266.0 MSL) for three seasons (Kharif-2017, Kharif-2018, and Kharif-2019), at CCS-HAU, Bawal (28°07′N, 77°10′E, 288.0 MSL) and at RARI, Jaipur (26°50′N, 75°47′E, 390.0 MSL) for a single season (Kharif-2019). Weather parameters from each environment during tillering to hard dough stage of plant [30–60 days after sowing (DAS)] are presented in Table 1. All climatic parameters except rainfall are presented as means over the crop-critical growing period 30–60 DAS. Rainfall is measured as cumulative rainfall received in mm and the cumulative number of rainy days. A canonical correspondence analysis (CCA) was performed to assess the impact of various environmental factors on FB score. The climatic determinants used for CCA includes the following: maximum and minimum, temperature (Max. Temp and Min. Temp), percentage maximum and minimum, relative humidity (Max. RH and Min. RH), and cumulative rainfall and rainy days (cumulative no. of days when daily rainfall measure above 2.5 mm). Weather data for the present analysis were obtained from the Division of Agricultural Physics, ICAR-IARI, New Delhi, India, and AICRP on Agro-meteorology, Hyderabad. The analysis was carried out in R package-“vegan”.

**TABLE 1:** Geographical identity and climate variables at test environment in respect of temperature, relative humidity (RH), total rainfall, and rainy days along with mean foliar blast (FB) score during the critical period of pearl millet crop growth.

Environment	Latitude	Longitude	Altitude	Max. temp. (°C)	Min. Temp. (°C)	Rainfall (mm)	Max. RH (%)	Min. RH (%)	Rainy days	Mean blast score
New Delhi-17	28.7	76.6	266	33.9	25.2	164.2	92.5	71.5	9.0	4.57
New Delhi-18	28.7	76.6	266	32.0	21.8	188.0	89.4	64.8	11.0	4.30
New Delhi-19	28.7	76.6	266	33.8	25.2	9.0	87.7	62.3	2.0	4.51
Jaipur-19	26.5	75.5	390	32.8	23.2	105.0	82.2	58.4	6.0	4.45
Bawal-19	28.1	77.1	288	33.0	23.6	28.0	90.0	55.4	1.0	4.48

### Agronomic Practices, Disease Screening, and Data Recording in Multi-Environment Field Trials

Each genotype was sown in a plot of two rows each of 2-m length having 65-cm row spacing and a 12-cm plant-to-plant spread. The sites for the research were carefully chosen based on the prevalence of *Magnaporthe grisea*. Spreader rows of FB-susceptible check (ICMB 95444) were planted after every 10th treatment of the test populations, and five rows of the spreader row of the susceptible check were planted on all the sides of the experimental area for maintaining sufficient disease pressure under natural condition. For a normal and healthy crop, standard cultivation practices recommended for pearl millet were regularly followed. FB scores were recorded from five randomly selected representative plants of all the genotypes in each replication, while days to 50% flowering (DFF) were recorded on a plot basis. Standard statistical methods were followed for data analysis. The disease was assessed following the 0–9 scale of Prakash et al. (2016) and Nayaka et al. (2017) as described earlier. The GE table of FB mean scores is transformed by subtracting each mean score from 9. Thus, the new score obtained as a consequence of this transformation adopted the same general interpretability principles as yield and other related traits, in which high values are preferred. The genotypes screened were categorized into five groups based on disease scoring: highly resistant (9), resistant (6–8), moderately resistant/susceptible (4–5), susceptible (2–3), and highly susceptible (0–1).

### Statistical Analysis

#### Variance Components and Estimation of Broad-Sense Heritability

Individual and combined analyses of variance (ANOVAs) were conducted on replicated data obtained in different environments (a combination of locations and years). The restricted maximum likelihood (REML) analysis was carried out for each environment, with replications as a fixed effect and genotypes and blocks within replication as random effects, while environments and replications within environments were considered as fixed effects whereas genotypes and genotype interactions with environments were considered as random effects in the REML model for combined environment analysis. The error variances of individual environments (a combination of locations and years) were accounted for combined analysis using the mixed model methodology. Error variance modeling using mixed model analysis takes care of heterogeneous error variances of the individual environment during pooled analysis. The REML (Patterson and Thompson, 1971) estimation technique was used to estimate three variance components (σ^2^_g_, σ^2^_ge_, and σ^2^_e_) for transformed FB score and DFF using the lmer function of the lme4 R-package (Bates et al., 2015). The rand function of the lmerTest package used the likelihood ratio test (LRT) at 5% probability to confirm the significance of the random effects (Kuznetsova et al., 2015).

The phenotypic observations *Y*_ijkm_ on genotypes *m* in replicate *j* of block *k* of environment *i* was modeled as follows:

*Y*_ijkm_ = μ + *e*_i_ + *r*_ij_ + *b*_ijk_ + *g*_m_ + (*ge*)*_im_* + (*eg*)*_jm_* + ε*_ijkm_*

where μ is the grand mean; *e*_i_ is the fixed effect of environment *i*; *g*_*m*_ is the random effect of genotype *m* and is ∼NID (0, σ_g_^2^); *r*_ij_ is the fixed effect of replication in the environment *i*; *b*_ijk_ is the random effect of block *k* nested with replication *j* in the environment *i* and is ∼NID (0, σ_b_^2^); (*ge*)*_im_* is the random effect of the interaction between genotype *m* and environment *i* and is ∼NID (0, σ_ge_^2^); ε*_ijkm_* is the random effect of the error variances.

Broad-sense heritability (H^2^) for the traits in each environment and over combined environments was estimated from the variance components. For each environment, H^2^ was calculated as H^2^ = σ_g_^2^/ (σ_g_^2^ + σ_e_^2^/r); and for combined environments, H^2^ was used as a measure of the trial’s reliability in genotype evaluation in this study, with H^2^ = 0 indicating that variations in genotypic mean in the trial are entirely attributable to random error and H^2^ = 1 indicating that differences are entirely due to genetic effects (Yan and Holland, 2010).

The REML model also produced the best linear unbiased predictors (BLUPs) of each genotype, thereby adjusting the influence of the neighboring rows. These BLUPs were used for downstream analysis.

#### Heritability-Adjusted Genotype Plus Genotype × Environment Interaction Biplot Analysis

Best linear unbiased predictors values of the transformed disease mean score were stored in a 250 genotypes × five environments matrix M. The matrix was checked for missing data arising due to non-germination of seed in the individual environment and was corrected through imputation using the expectation–maximization algorithm implemented by R package, bbplot/R Bilinear as suggested by Gauch and Zobel (1990). Furthermore, heritability-adjusted scaling (Yan and Holland, 2010) was performed in R. The entries that were identified as resistant were further highlighted in GGE biplot construction for better visualization.

The GGE biplot was constructed by estimating each element of the matrix using the following formula, based on the first two principal components (PCs) resulting from singular value decomposition (SVD) (Yan et al., 2000; Yan and Kang, 2003):

$$\text{Yij}=\mu +\text{ej}+\sum_{n=1}^{\text{N}}\lambda_{\text{n}}\gamma_{\text{in}}\delta_{\text{jn}}+\varepsilon ij.$$

Where,

Y_ij_ = mean response of i^th^ genotype (i = 1, …, i) in the j^th^ environment (j = 1, …, j);

μ = grand mean;

e_j_ = environment deviations from the grand mean;

λ_n_ = the eigenvalue of PC analysis axis;

γ_in_ and δ_jn_ = genotype and environment PC scores for axis n;

N = number of PCs retained in the model;

ε_ij_ = residual effect ∼ *N*(0, σ^2^_e_).

An “average environment coordination” (AEC) view of the GGE biplot was used to appraise genotypic response and stability. It facilitated genotype comparisons based on disease score mean and stability across environments within a “mega-environment” (Yan, 2001, 2002). The axis of the “AEC abscissa,” denoted by a single arrowed line, indicated higher mean performance of genotypes in terms of higher FB resistance, whereas the “AEC ordinate,” denoted by a line perpendicular to the AEC abscissa and passing through the origin of the biplot, represented genotype stability. Stability is represented by projections on the AEC abscissa connecting individual genotypes (Yan and Falk, 2002). Similarly, the “discriminating power vs. representativeness” view of the GGE biplot was constructed for the evaluation of test environments, where the “ideal” test environment should be both discriminating with respect to genotypes and representative of the “mega-environment” (Yan et al., 2007). In addition, a “desirability index” of the test locations has been compiled taking into account the relationship between the test environments and distance from the ideal genotype (Yan and Holland, 2010). Angles between the various environment vectors were used to judge the correlation among the environments in order to determine the relationship between test locations (Yan and Kang, 2003). Furthermore, a “which-won-where” view of the GGE biplot has been prepared to determine the superiority of the genotypes in different test environments as well as grouping of test environments into different “mega-environments” (Yan and Rajcan, 2002). Finally, bootstrapping (re-sampling process is repeated 10,000 times to obtain 10,000 bootstrap samples) is used to assess the validity of GGE biplot. These bootstrap samples were later used to construct CL at the 95% level for individual genotype and environment PC scores as suggested by Hu and Yang (2013). The raw data with columns representing the environments (*p* = 5) and rows representing the genotypes (*n* = 43) were later average-centered for each environment, resulting in a mean of zero for each of the p dimensions of raw data. The data were subjected to bidirectional bootstrapping and procrustes rotation in the R package “bbplot/R,” and the confidence regions were computed using a distribution-free approach implemented in the R package “distfree.cr/R” based on the empirical distribution of the aligned genotypic or environmental scores from all bootstrap samples.

## Results

The diversity panel of 250 accessions along with checks was subjected to phenotyping for FB incidence and DFF at five different environments. Phenotypic data collected from the population at three different locations during the rainfed seasons of 2017, 2018, and 2019 were statistically analyzed to determine variance components. Hereafter, the five environments are denoted as ND-17 (New Delhi-17), ND-18 (New Delhi-18), ND-19 (New Delhi-19), BWL-19 (Bawal-19), and JPR-19 (Jaipur-19).

### Identification of Climatic Factors Influencing Foliar Blast Infection

**FIGURE 2:**
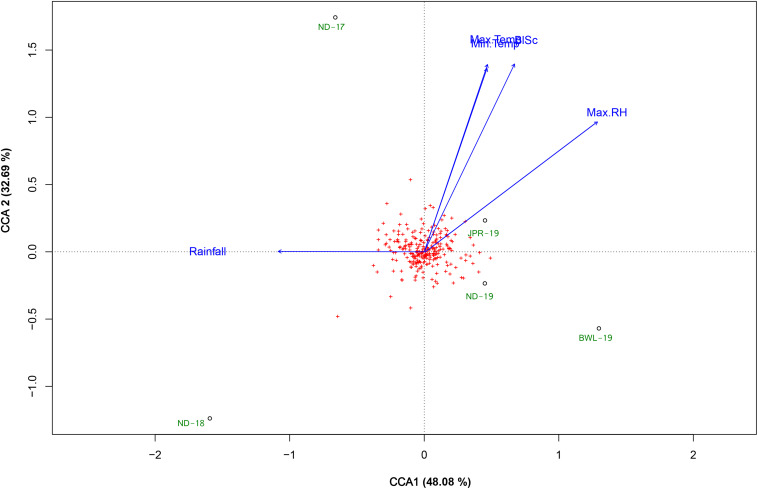
Canonical correspondence analysis (CCA) biplot based on the correlation of several climatic parameters with foliar blast score recorded from a set of a diversity panel of 250 pearl millet accessions studied in five environments (ND-17, New Delhi-2017; ND-18, New Delhi-2018; ND-19, New Delhi-2019; BWL-19, Bawal-2019; JPR-19, Jaipur-2019). The climatic determinants were recorded during the critical crop growing period 30–60 days after sowing (DAS) period, as follows: Max. Temp, maximum temperature (°C); Min. Temp, minimum temperatures (°C); Max. RH, maximum relative humidity (%); Min. RH, minimum relative humidity (%); Rainfall, cumulative rainfall (mm); RD, cumulative number of days on which received daily rainfall > 2.5 mm; BlSc, foliar blast score.

Weather parameters observed in each environment during tillering to hard dough stage of plant growth (30–60 DAS) are presented in Table 1, and their influence over FB score is elucidated in the CCA diagram (Figure 2). CCA biplot explained 80.77% of the total variation between the site weather parameters and the FB score. The first CCA axis explained 48.08%, and the second CCA axis explained 32.69% of the total variation. Maximum temperature (Max. Temp), minimum temperature (Min. Temp), and maximum humidity (Max.RH) were the main determinants and were positively associated with the increase of FB score during the critical growth period of the crop. Cumulative rainfall recorded during the critical period of growth of the crop was not associated with FB score. Based on spatial angular proximity of identified climatic determinants (Max. Temp, Min. Temp, and Max. RH), JPR-19 was found to be conducive for FB disease in pearl millet.

### Mean Performance of Genotypes and Analysis of Variance

**FIGURE 3:**
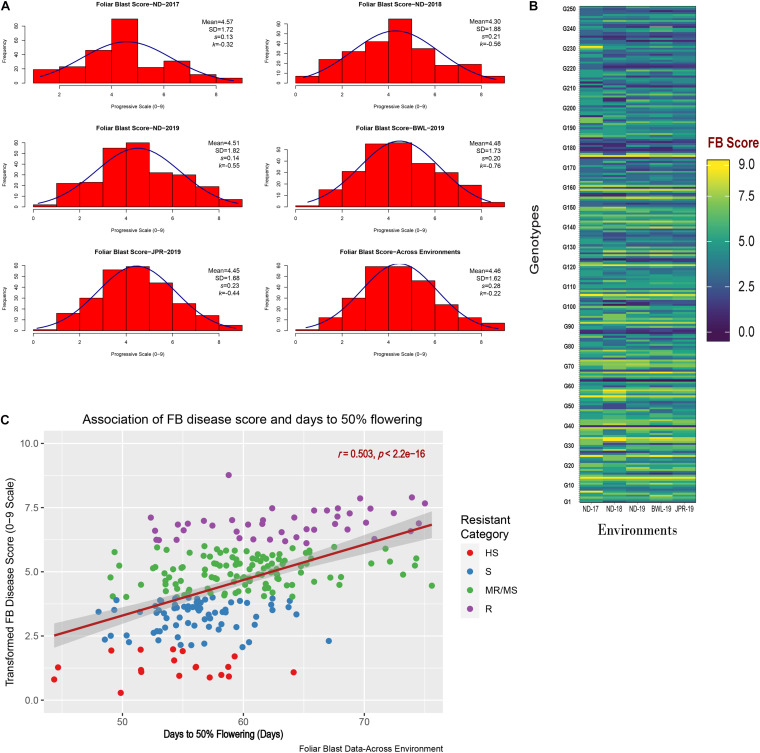
**(A)** Frequency distribution for foliar blast (FB) scores in five different environments along with overall distribution indicating FB scores across the environment. **(B)** Heatmap visualization of genotypic FB scores observed in individual environments. Environments are depicted in the x-axis, whereas the genotypes are depicted in the y-axis containing the corresponding mean FB score recorded. **(C)** Scatter plot representing the association of FB disease scores (0–9 scale) with corresponding days to 50% flowering (in days) of 250 genotypes observed across environments. Days to 50% flowering of genotypes are depicted in the x-axis, whereas the transformed FB scores of genotypes are depicted by the y-axis. The regression line (with 95% confidence level) is indicated by brick red lines with gray shadows. The color of the dots indicates the resistant category to which the genotypes belong.

The experiment was executed systematically in all the five environments. Susceptible check, ICMB 95444, included in the experiment exhibited uniform FB-susceptible reaction indicating the availability of sufficient inoculums for disease screening. The mean value, standard deviations, and the frequency distribution for FB scores within and across years and locations indicate that the lines exhibited similar levels of disease severity in all five environments (Figure 3A) although slight differences in distribution are evident from the histogram for ND-17. The average FB score in ND-17 was marginally higher than the average scores, but the distribution pattern was flatter, indicating a higher level of variability (SD = 1.7). ND-18 showed the highest variability (SD = 1.88), yet the distribution was slightly skewed toward the resistant side, with FB score considerably lower than the remaining four environments. Moreover, there was a presence of crossover G × E interaction among genotypes for FB scores, which were evident from the heatmap visualization of the GE interaction (Figure 3B). From the pooled data, out of 250 genotypes, none of the genotypes showed a highly resistant reaction (score = 9) to FB. However, at the hard dough stage, 43 genotypes were reported as resistant (R), 118 as moderately resistant/susceptible (MR/MS), 70 as susceptible (S), and 19 as highly susceptible (HS) to FB (Figure 3C). Out of 43 resistant lines, five were late in flowering (51–54 days), whereas 38 lines flowered very late (>54 days). Among 43 resistant lines, 26 genotypes exhibited a resistance reaction above the qualifying check, ICMR 11009 (score = 6.5), across environments (Table 2). Also, five more genotypes have an FB score that is at par with the qualifying check. Genotypes IP 11353, IP 22423, IP 7941, IP 7910, IP 12322, and IP 3106 were consistently showing higher resistance and outperformed the corresponding qualifying checks identified for each environment (indicated in blue color). Considering the frequency of appearance in the top 10 lines based on FB scores, only three genotypes, namely, IP 11353 (G-62), IP 22423 (G-39), and IP 7941 (G-220), were found to be more consistent with the lowest stability index below 0.1 in all the five test environment.

**TABLE 2:**
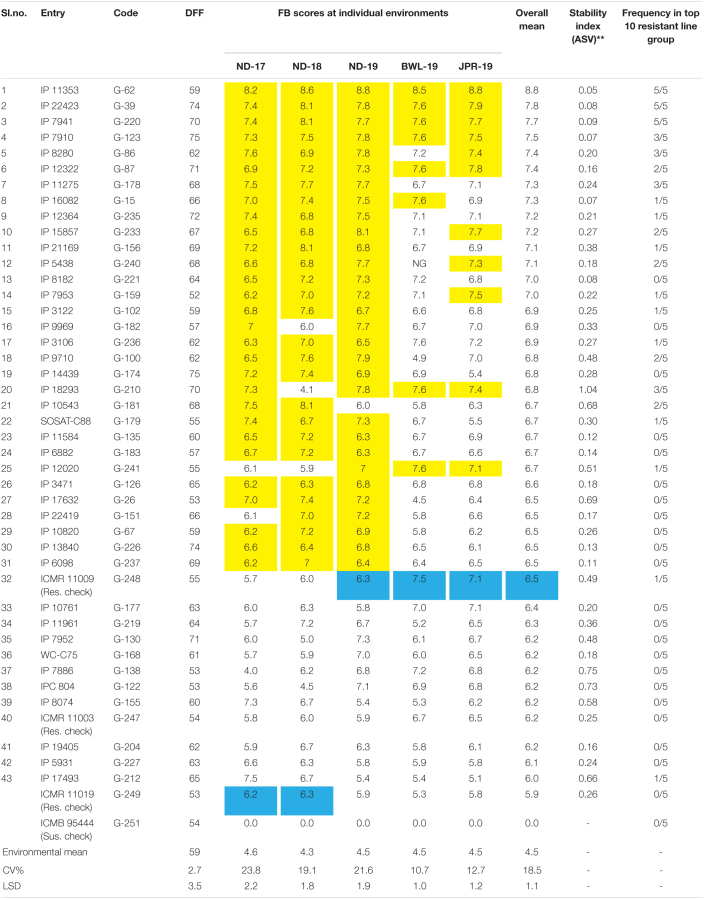
List of genotypes outperforming resistant checks for foliar blast resistance across environments.

*Yellow color represents genotypes whose foliar blast score is above qualifying check. *Blue color represents qualifying checks under each environment. **Stability index worked out as described by Purchase et al. (2000); ASV = AMMI stability value (stable genotypes will have lower values). CV, coefficient of variation; LSD, least square difference; DFF, days to 50% flowering; NG, seeds not germinated; ND-17, New Delhi-2017; ND-18, New Delhi-2018; ND-19, New Delhi-2019; BWL-19, Bawal-2019; JPR-19, Jaipur-2019.

### Analysis of Variance and Estimation of Heritability

Analyses of variance of individual environment indicated significant genotypic differences (*p* < 0.001) for DFF and FB disease score at the hard dough stage. FB disease score has shown the highest genotypic variance at ND-18, and DFF has shown the highest variance at BWL-19 (Table 3). Combined ANOVA also revealed significant genotypic differences along with significant environment and GEI effects (*p* < 0.001) for DFF and FB disease score at the hard dough stage. The contribution of genotypic variance toward total phenotypic variance was higher for FB disease score, whereas the contribution of GEI variances was higher compared with genotypic variance for DFF (Tables 3, 4). The contribution of environment variance was very low for both DFF and FB disease scores. Probability plots of residuals *versus* expected values indicated no discernible trend, implying that the assumptions of independence and equal variance were fulfilled.

**TABLE 3:** Analysis of variance for foliar blast score and days to 50% flowering at individual environment under rainfed conditions.

Traits	Effects	Source of variance	DF	New Delhi-17				New Delhi-18				New Delhi-19				Bawal-19				Jaipur-19			
				Variance	SE	F value^a^ and LRT value^b^	*p* values	Variance	SE	F value^a^ and LRT value^b^	*p* values	Variance	SE	F value^a^ and LRT value^b^	*p* values	Variance	SE	F value^a^ and LRT value^b^	*p* values	Variance	SE	F value^a^ and LRT value^b^	*p* values
Foliar blast score	Fixed	Replication	1	–	–	0.10	0.758	–	–	0.24	0.628	–	–	0.005	0.945	–	–	0.06	0.810	–	–	0.291	0.597
	Random	Genotype	249	2.89	1.7	165.70	<0.001	3.616	1.9	263.12	< 0.001	3.46	1.86	430.44	<0.001	3.40	1.85	230.45	<0.001	2.92	1.71	481.032	<0.001
		Block (Replication)	48	0.06	0.25	1.55	0.213	0.011	0.11	0.11	0.743	0.04	0.19	4.56	0.033	0.02	0.12	0.14	0.707	0.01	0.07	0.213	0.644
		Residuals	201	1.16	1.1		<0.001	0.841	0.92		<0.001	0.32	0.57		<0.001	0.95	0.98		<0.001	0.23	0.48		<0.001
	Heritability			0.833				0.896				0.955				0.877				0.961			
Days to 50% flowering	Fixed	Replication	1	–	–	0.34	0.572	–	–	0.036	0.852	–	–	0.45	0.508	–	–	1.43	0.255	–	–	0.009	0.925
	Random	Genotype	249	47.86	6.9	717.77	<0.0001	34.728	5.893	393.480	<0.001	62.79	7.92	570.86	<0.001	68.37	8.27	1,188.93	<0.001	59.12	7.69	535.730	<0.001
		Block (Replication)	48	0.00	0.0	0.00	1.000	0.032	0.180	0.032	0.858	0.25	0.50	2.57	0.109	0.00	0.00	0.00	1.000	0.45	0.67	5.532	0.019
		Residuals	201	1.30	1.1		<0.001	4.179	2.044		<0.001	3.19	1.79		<0.001	0.28	0.53		<0.001	3.38	1.84		<0.001
	Heritability			0.987				0.943				0.975				0.978				0.972			

*DF, degree of freedom; SE, standard error. ^a^Concerning the fixed effect components. ^b^Concerning the random effect components.*

**TABLE 4:** Combined analysis of variance for foliar blast resistance and days to 50% flowering in pearl millet.

Traits	Effects	Source of variance	DF	Variance	SE	F value^a^ and LRT value^b^	*p* values	H^2^
Foliar blast score	Fixed	Environments	4			6.017	<0.0001	
		Environments (Replication)	5			0.191	0.966	
	Random	Genotype	249	2.792	1.671	1,106.751	<0.0001	0.887
		Genotype × Environments	996	0.474	0.689	192.564	< 0.0001	
		Environments (Replication × Block)	240	0.015	0.124	2.057	0.151	
		Residuals	1,245	0.709	0.842			
Days to 50% flowering	Fixed	Environments	4			179.725	<0.0001	
		Environments (Replication)	5			0.256	0.937	
	Random	Genotype	249	38.105	6.173	811.343	<0.0001	0.968
		Genotype × Environments	996	16.438	4.054	1,610.839	<0.0001	
		Environments (Replication × Block)	240	0.093	0.305	3.160	0.075	
		Residuals	1,245	2.521	1.588			

*DF, degree of freedom; SE, standard error; H^2^, heritability. ^a^Concerning the fixed effect components. ^b^Concerning random effect components.*

In the current analysis, both traits were strongly heritable (>0.60) in individual environments, as per the Robinson et al. (1966) scale of heritability (Table 3). Compared with FB disease score, DFF was found to be more heritable. Broad-sense heritability for both the traits was also higher (>0.60) across five environments (Table 4). However, when assessed on the basis of pooled environment, a partitioning of GEI component lowered heritability for both the traits across environments. For foliar disease score, broad-sense heritability ranged from 0.83 (ND-17) to 0.96 (JPR-19); and in the pooled environment analysis, it was 0.89. Similarly, for DFF, it ranged from 0.94 (ND-18) to 0.99 (BWL-19) in case of the individual environment, while in case of pooled data, heritability estimate was 0.97. High heritability for both the traits indicated that the genotypic differences observed are mainly due to genetic effects. Also, there was a moderate, significant positive correlation between FB disease score (transformed data) and DFF (*r* = 0.503, *p* < 0.0001), indicating that FB disease resistance in pearl millet is associated with very late flowering (Figure 3C).

### Estimation of Best Linear Unbiased Predictor Values and Imputation of Missing Data

**FIGURE 4:**
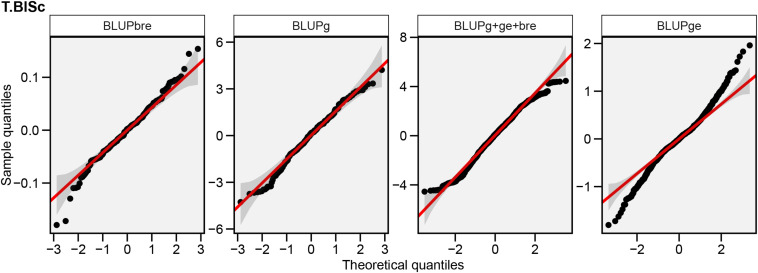
Normal Q-Q plots showing the distribution of random effects associated with block within replication (BLUPbre), genotype (BLUPg), total phenotypic effects (BLUPg + ge + bre), and GEI (BLUPge) depicting 95% confidence level for adjustment to normality recorded across environments.

Predicted genetic values (BLUP) were estimated to guide the inferences based on a multi-environment GGE model with reduced biases arising from uncontrollable factors (Figure 4). It was useful for recommending genotypes with the minimum likelihood of error while recommending them for a specific environment. For the individual environments, the predicted FB score ranged from 0.81 to 8.21 in ND-17, from 0.48 to 8.56 in ND-18, from 0.19 to 8.79 in ND-19, from 0.61 to 8.50 in BWL-19, and from 0.29 to 8.84 in JPR-19. Under all the environments, genotype G-13 (IP 4020) had the lowest resistance score, and genotype G-62 (IP 11353) had the highest level of FB resistance. Similarly, across the environment, the estimate of genotype (random effect) for FB score ranged from 0.28 (G-13) to 8.77 (G-62). Prior to heritability scaling, the genotype–environment BLUP matrix (M) was analyzed for missing data. It was observed that around 1% of missing data were solved by imputation by the expectation–maximization algorithm.

### Detection of Ideal Genotype Based on Mean *Versus* Stability

**FIGURE 5:**
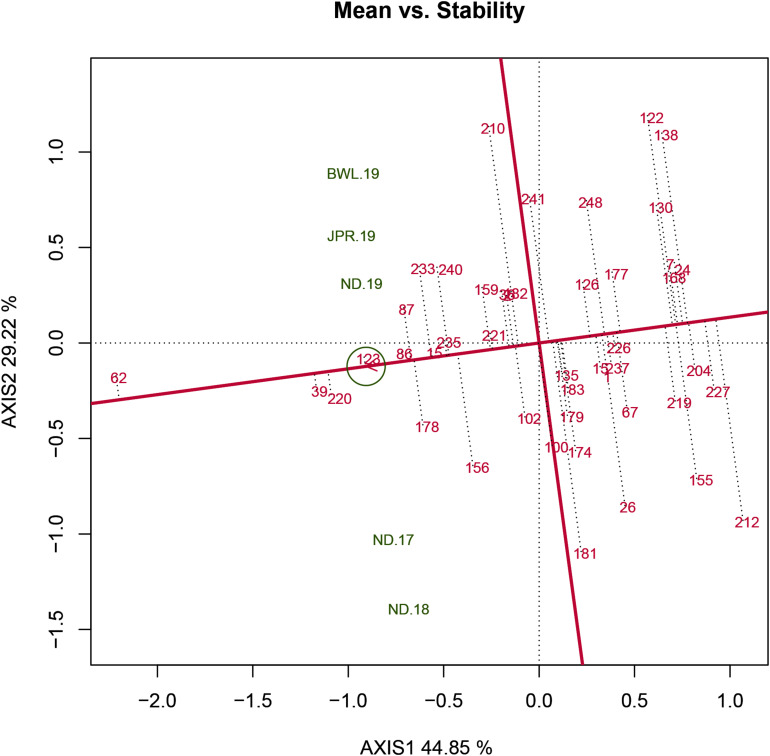
Mean *vs* stability view of the GGE biplot of 43 resistant genotypes across five testing environments. There was heritability-adjusted scaling of data where the environment standardized data were multiplied by the heritability in each environment (transform = HA), and data were centered by means of the environments (centering = 2). The biplot was based on “row metric preserving” [singular value partition (SVP) = 1], which means that the singular values were partitioned into the genotype eigenvectors for visualizing the correlation among genotypes. Numbers correspond to genotypes as listed in Supplementary Table 1. Environment: ND-17, New Delhi-2017; ND-18, New Delhi-2018; ND-19, New Delhi-2019; BWL-19, Bawal-2019; JPR-19, Jaipur-2019.

**FIGURE 6:**
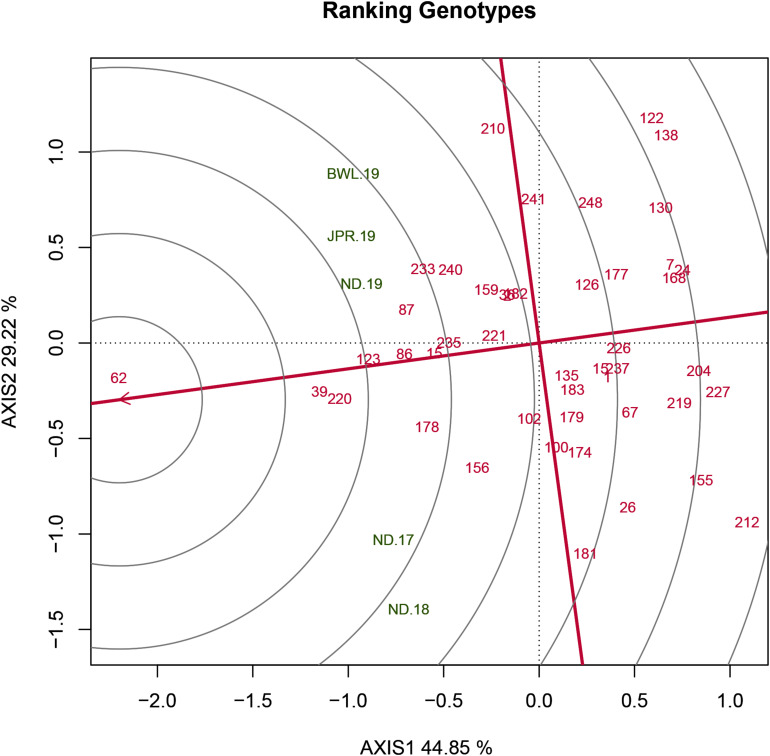
Genotype ranking plot view of the GGE biplot of 43 resistant genotypes across five testing environments. There was heritability-adjusted scaling of data where the environment standardized data were multiplied by the heritability in each environment (transform = HA), and data were centered using the environments (centering = 2). The biplot was based on “row metric preserving” (SVP = 1), which means the singular values were partitioned into the genotype eigenvectors for visualizing the correlation among genotypes. Numbers correspond to genotypes as listed in Supplementary Table 1. Environment: ND-17, New Delhi-2017; ND-18, New Delhi-2018; ND-19, New Delhi-2019; BWL-19, Bawal-2019; JPR-19, Jaipur-2019.

**FIGURE 7:**
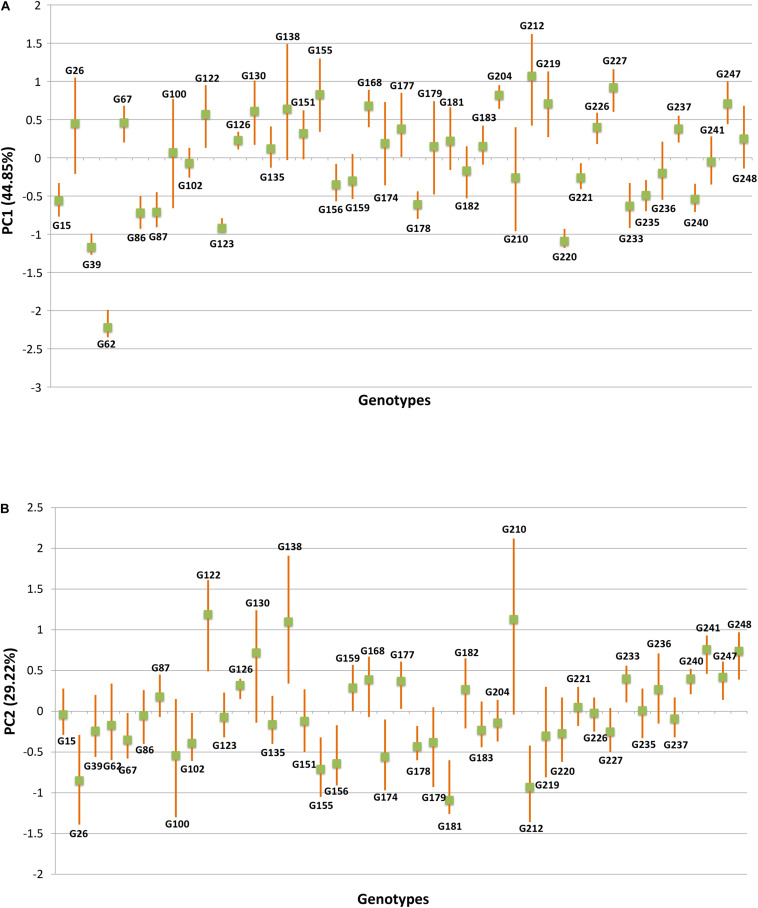
The fitted principal components (PCs) *versus* genotype based on foliar blast disease score along with upper and lower limit values, produced by bootstrap analysis over 10,000 times at 95% bias-corrected and accelerated bootstrap (BCa) confidence limits (CLs). **(A)** Principal component analysis (PCA) score values on PC1 *versus* genotype. **(B)** PCA score values on PC2 *versus* genotype.

The HA-GGE biplot is the most precise way to detect ideal genotypes. An ideal genotype should have both the highest mean performance and the lowest interactions with the environment. The mean *vs* stability biplot view of the HA-GGE biplot was generated based on the principle of genotype-focused singular value partition (SVP) (SVP = 1) as suggested by Yan (2002). This biplot view portrays the ranking of genotypes based on their average FB score across environments (Figure 5). PC 1 and PC 2 explained 44.85 and 29.22%, respectively, of the total variation of the environment scoring. The single arrowhead line passing through the origin, the AEC abscissa, indicated a highly resistant genotype with a lower FB score. Therefore, genotypes positioned downstream of the arrow are considered promising for FB resistance reaction. IP 11353 (G-62), IP 22423 (G-39), IP 7910 (G-123), and IP 7941 (G-220) were positioned downstream of the biplot origin and, therefore, experienced less FB score. Moreover, the stability of the genotype could be accessed through the length of the projection in both directions from the AEC abscissa, that is, the AEC ordinate. Thus, if the genotype had greater projection from the AEC abscissa, it would be less stable. Considering both mean performance and stability, IP 11353 (G-62) was the ideal genotype, having less disease score and high stability (Figure 6). Since the distance between two genotypes should always be estimated by Euclidian distance, genotypes that are closer to the ideal genotype are considered to be desirable (Yan and Tinker, 2006). Therefore, IP 22423 (G-39), IP 7910 (G-123), and IP 7941 (G-220) were identified as desirable genotypes with lesser FB scores and almost consistent performance. Furthermore, using the CL at the 95% level for individual genotypic scores on FB as well as environmental scores corresponding to PC 1 and PC 2 (Supplementary Table 2), bootstrapping revealed that PC 1 contributed more to the significant differences among genotypes, as seen on the biplot (Figure 7). In terms of FB scores, it was established that the ideal genotype IP 11353 (G-62) was statistically different from the three desirable genotypes, IP 22423 (G-39), IP 7910 (G-123), and IP 7941, based on PC 1 scores (lower limit, 2.35; and upper limit, 1.99) (G-220). Three desirable genotypes, on the other hand, showed no significant differences in their PC 1 scores for both parameters.

### Evaluation of Testing Locations Based on Discrimination Power *Versus* Representativeness and Desirability Index

**FIGURE 8:**
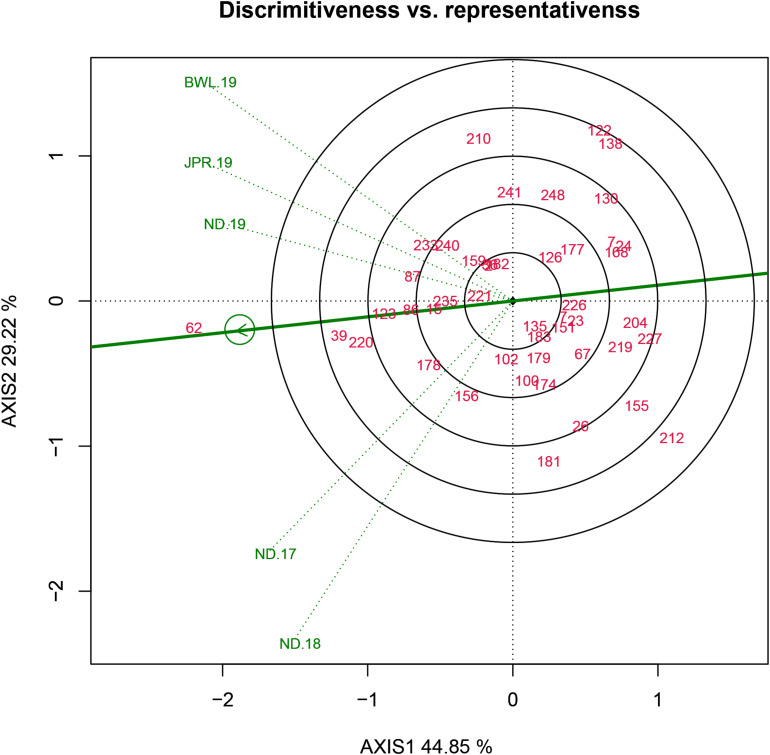
Discriminativeness *vs* representativeness view of test locations based on GGE biplot of 43 resistant genotypes across five testing environments. There was heritability-adjusted scaling of data where the environment standardized data were multiplied by the heritability in each environment (transform = HA), and data were centered by means of the environments (centering = 2). The biplot was based on “Column metric preserving.” Numbers correspond to genotypes as listed in Supplementary Table 1. Environment: ND-17, New Delhi-2017; ND-18, New Delhi-2018; ND-19, New Delhi-2019; BWL-19, Bawal-2019; JPR-19, Jaipur-2019.

Three parameters, namely, discrimination power (ability to segregate the tested genotypes), representativeness (ability to represent the ME) and desirability index (the joint response of both discriminating power and representativeness) are crucial for assessing the test environment in the GGE biplot approach. In the HA-GGE biplot, the length of the environmental vector, which is approximately the square root of heritability, represents discrimination ability. The angle between the environmental vectors and the AEC abscissa specifies the representativeness of the testing location. The environment becomes more representative when the angle among the test environment with AEC abscissa becomes more acute. In analysis, it was found that among the test environments, BWL-19 had the longest environmental vectors, rendering it as the most “discriminating location” with the ability to discriminate genotypes from other sites. However, in the case of representativeness, ND-19 showed a minimum angle with average environment followed by JPR-19. Hence, the desirability index was worked out to identify the most ideal testing location accounting for both discrimination ability and representativeness (Table 5). The Jaipur center having the highest desirability index (1.026) was identified as an ideal or a type I testing location for testing of a mini core collection or advance breeding materials against FB disease. Since ND-19 had also been included in the same sector, therefore, it can be considered as a supplementary or type II location for testing pearl millet genotypes against FB (Figure 8).

**TABLE 5:** Standardized test environment evaluation parameters.

Environment	Discriminative	Representativeness	Desirability index	Desirability index rank
ND-17	1.257	0.771	0.969	4
ND-18	1.446	0.629	0.910	5
ND-19	1.100	0.932	1.025	2
BWL-19	1.361	0.750	1.021	3
JPR-19	1.187	0.864	1.026	1

### Relationship Among Environments and Mega-Environment Delineation

**FIGURE 9:**
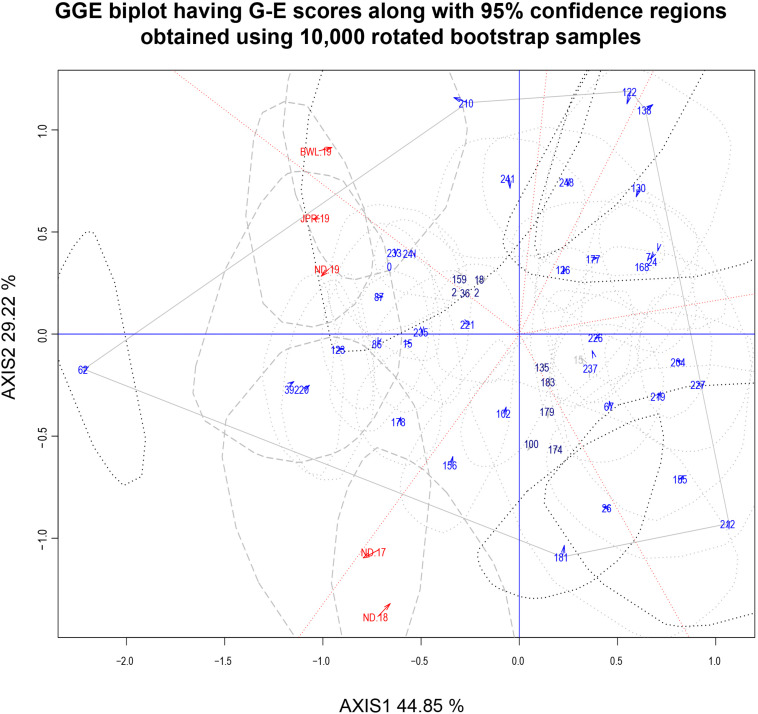
Which-won-where biplot view of 43 genotypic scores and five environmental scores constructed along with the 95% confidence regions using 10,000 rotated bootstrap samples. There was heritability-adjusted scaling of data where the environment standardized data were multiplied by the heritability in each environment (transform = HA), and data were centered by means of the environments (centering = 2). The biplot was based on “row metric preserving” [singular value partition (SVP) = 1], which means that the singular values were partitioned into the genotype eigenvectors for visualizing the correlation among genotypes. Numbers correspond to genotypes as listed in Supplementary Table 1. Environment: ND-17, New Delhi-2017; ND-18, New Delhi-2018; ND-19, New Delhi-2019; BWL-19, Bawal-2019; JPR-19, Jaipur-2019.

In the current study, “which-won-where” biplot for FB score created a hexagon with six genotypes, G-62, G-210, G-122, G-138, G-242, and G-181, at the vertices (Figure 9). The equality lines divided the polygon into six sectors effectively. Five testing environments were spread in two sectors within the biplot: three in one and two in another. This illustrated that the testing locations could be divided into MEs. The first ME (ME-I) was represented by ND-19, JPR-19, and BWL-19, with IP 11353 (G-62) having the highest FB resistance as the winning genotypes. The second ME (ME-II) was composed of ND-17 and ND-18 having IP 10543 (G-181) as the winning genotype. All environments within the MEs exhibited acute angles, resulting in a positive association with each other. Genotypes IP 8182 (G-221), IP 11584 (G-135), IP 6883 (G-183), IP 16082 (G-15), IP 6098 (G-237), and IP 13840 (G-226) were placed near to the origin depicting consistency in the performance.

## Discussion

The FB of pearl millet is a severe menace to successful pearl millet cultivation in the Indian subcontinent particularly in A_1_ and A zones, which causes considerable yield losses of both grain and forage (Adhikari et al., 2020). A few genotypes with partial resistance to the disease have been identified (Thakur et al., 2009, 2011; Sharma et al., 2013, 2020; Goud et al., 2016; Prakash et al., 2016). Since FB is controlled by a few genetic loci with immense environmental influence, progress through conventional breeding has been very limited. Moreover, the rapid emergence of new isolates and the complex nature of host–pathogen interaction along the confounding environmental effect have made it difficult to pinpoint the various sources of durable resistance (Adhikari et al., 2020; Sharma et al., 2020). Therefore, in this study, the HA-GGE biplot approach was used as an effort to identify durable FB resistance sources in pearl millet with little GEI effect and having a genetic buffering capacity to overcome the pathogenic attack.

In the present study, genotypes showed significant differences for both DFF and FB incidence in all five environments. Even though mean FB incidence was almost similar for all the five environments, ND-17 showed a slightly higher incidence followed by ND-19 and BWL-19. With respect to environment variables, it was observed that maximum temperature (Max. Temp), minimum temperature (Min. Temp), and maximum relative humidity (Max. RH) were slightly higher at ND-17 followed by ND-19. These factors might have played a significant role in determining the emergence of the blast disease as also reported by Pattanayak and Das (2020).

The G, E, and G × E interactions displayed significant differences as revealed by ANOVA. The presence of a significant G × E interaction indicated that the FB incidence of tested genotypes varied across environments, which could be attributed to different agro-ecologies with varying longitude, latitude, and elevation. Significant GEI also suggested the need to develop FB-resistant lines with specific adaptation to target ecology. Furthermore, genotypic variance contributed more to disease resistance than the G × E relationship, suggesting that genetic variation accounted for the most of the variation in disease reactions. Persaud and Saravanakumar (2018) also reported greater contribution of genotypic factor over G × E interaction factor while conducting a multilocation experiment in case of rice FB in which 76.02% of the total SS was attributed to genotype (G) effect, 3.10% to environment (E) effects, and 20.88% to GEI effects. Similar results were also reported by Beyene et al. (2012) in maize foliar disease resistance, Sharma et al. (2012) in chickpea wilt incidence, and Sharma et al. (2016) in pigeon pea–*Fusarium udum* interaction.

An initial study of 250 genotypes facilitated the selection of 43 resistant genotypes for HA-GGE biplot analysis. The complex GEIs were simplified in different PCs and graphically presented against various PCs in GGE biplot analysis, and their contribution justified the GGE biplot’s utility in explaining sources of variation (Yan and Tinker, 2005). In the present study, the first two PCs clarified more than 70% of the total variance, indicating that the variability for FB resistance reaction is adequately represented. The “mean vs. stability view” of the biplot for the trait represented differential responses of tested genotypes to diverse environments due to the existence of crossover interactions. Genotype ranking in terms of resistance to blast was observed to change from one environment to another. The genotypes IP 11353 (G-62), IP 22423 (G-39), IP 7910 (G-123), and IP 7941 (G-220) were positioned downstream of the biplot origin and, therefore, experienced less FB score and were considered resistant. Among these genotypes, IP 11353 (G-62) was considered to be the ideal genotype owing to its higher disease resistance and smaller interaction with the environment in the form of a high projection from the AEC abscissa (Yan and Falk, 2002). Genotypes that are in proximity with “ideal” were considered “desirable” due to their high genetic relationship with the “ideal” genotype (Yan and Tinker, 2005). IP 22423 (G-39), IP 7910 (G-123), and IP 7941 (G-220) were identified as desirable genotypes owing to their proximity to the ideal genotypes that differ in their ability to respond to fungal infection by inducing long-lasting, broad-spectrum, and systemic resistance. Bootstrapping at 95% CL improved the precision of the visual observation recorded on promising genotypes. The ideal genotype revealed a significant statistical difference from the desirable genotypes. However, all of the desirable genotypes were overlapping. HA-GGE biplot has successfully detected stable and resistant genotypes in various crops (Silva et al., 2011; Sillero et al., 2017; Sánchez-Martín et al., 2017; Parihar et al., 2018; Singh et al., 2020). Thus, the “ideal” genotypes, along with any one of the “desirable” genotypes having durable resistance, would be valuable genetic resources in the upcoming comprehensive resistance breeding program of pearl millet.

Heritability-adjusted genotype plus genotype × environment interaction biplot identified the superior testing location, facilitating complete resource allocation with minimum trial cost without compromising trial heritability and genetic gain under selection (Yan, 2001; Yan and Tinker, 2006; Yan and Holland, 2010). Previous reports also claimed that, assuming adequate discriminating capacity, “representativeness” is the most important factor to be considered when deciding how a test location should be used in genotype evaluation (Yan et al., 2007). The square root of heritability (√H^2^) of each test environment based on vector length and the representativeness as its genetic correlation with other test environments (r) based on the angle between two test environments can be assessed by HA-GGE biplots (Allen et al., 1978; Flores et al., 2013). Considering both the parameters, Jaipur center (JPR-19), with the highest desirability index, was recognized as the ideal or type I testing location for testing advanced breeding materials for FB-resistant progenies during the early breeding stage. The existence of non-crossover GEI (consistent performance of genotype) suggested the presence of a close relationship among the test locations. Thus, HA-GGE biplot is the most precise method for proper delineation of an ideal testing location.

The only way to accomplish consistent genotype performance within a given sector is to divide testing locations into distinct “mega-environments.” “A mega-environment can be described as a group of analogous locations delivering similar genotypic responses when sharing the same set of genotypes across the year” (Yan and Rajcan, 2002). The “mega-environment” can be effectively depicted using a “which-won-where” view of GGE biplot methodology (Gauch and Zobel, 1997; Yan and Kang, 2003; Yan et al., 2007). The aim of ME diagnosis is to better understand the complex GEI pattern that occurs within that region in order to exploit specific adaptations and maximize selection responses (Yan, 2011). In the current investigation, the HA-GGE biplot was able to separate all of the testing locations into two distinct MEs to aid the restructuring of agro-ecological zones. Year 2019 was separated as single ME (ME-I) in which the most desirable environment JPR-19 was also included. Environmental conditions of the Jaipur location were found to be more conducive for FB incidence. Hence, Jaipur location owing to its informative role in the present study can be selected in future FB testing programs. Bootstrapping at 95% CL improved the precision of recommendation of a testing location and ME delineation. However, reported groupings of environments need to be further reconfirmed using multi-location testing data over more number of years as also reported by earlier authors (Yan et al., 2000; Silva et al., 2011; Phuke et al., 2017; Sánchez-Martín et al., 2017; Das et al., 2020). Also, the performance and stability of all selected materials advocate additional testing in central and southern pearl millet-growing regions of the country for the future development of elite FB-resistant cultivars.

## Conclusion

In the present study, genotypic effects and GEI exhibited the greatest effect in comparison with the environment alone for FB resistance in pearl millet. Based on the HA-GGE biplot, all of the tested locations could be grouped into two distinct MEs with winning genotypes. It confirms the presence of crossover-type GEI and emphasizes breeding for environment-specific adaptability. More importantly, among the tested genotypes, IP 11353 was recognized as “ideal,” and IP 22423, IP 7910, and IP 7941 were recognized as “desirable” genotypes, having stable resistance against the disease. Salient findings obtained in the present study were also validated by bootstrapping at 95% CL. This study was also able to reorganize delineated MEs and advocates precise testing of materials with optimization of resources in future breeding programs.

## Data Availability Statement

The raw data supporting the conclusions of this article will be made available by the authors, without undue reservation.

## Author Contributions

RS and SPS designed and supervised the overall research and contributed to the preparation of the manuscript. CS provided technical guidance and liaised among multi-environment locations. SMS, YY, and LS executed the field experiments. SMS and GP performed the phenotyping and disease scoring. AR and SLS carried out statistical analysis and preparation of the draft manuscript. SLS and NS performed manuscript review. RS edited the manuscript for final submission. All authors contributed to the article and approved the submitted version.

## Conflict of Interest

The authors declare that the research was conducted in the absence of any commercial or financial relationships that could be construed as a potential conflict of interest.
