# Supplementary figures and images for: Deciphering Genotype-By-Environment Interaction for Target Environmental Delineation and Identification of Stable Resistant Sources Against Foliar Blast Disease of Pearl Millet
Source: Front Plant Sci. 2021 May 17;12:656158. doi: 10.3389/fpls.2021.656158 (PMC8165241; doi:10.3389/fpls.2021.656158)

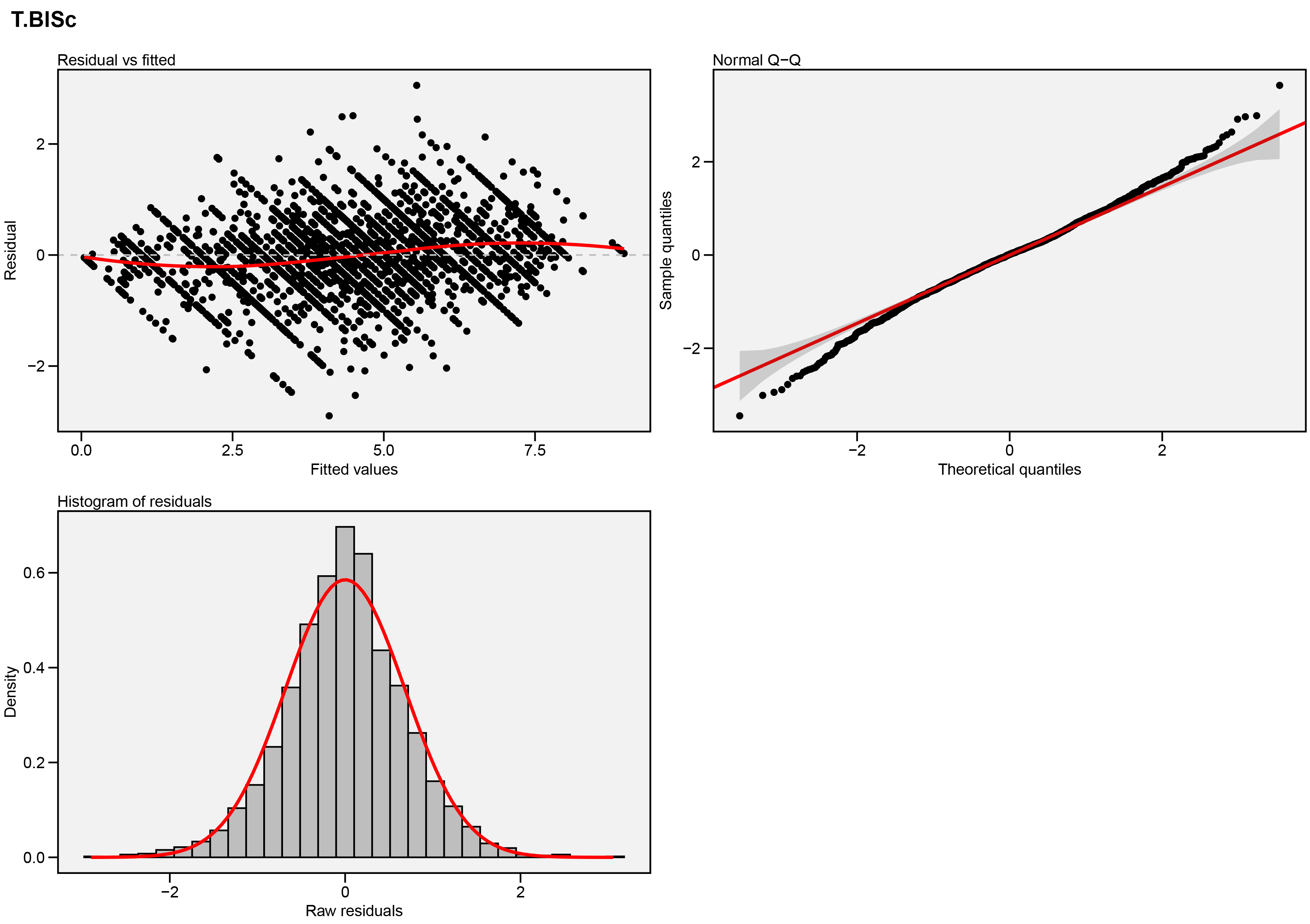

Supplement: Supplementary file 1 [file Image_1.TIF]
